# Applications of random forest feature selection for fine‐scale genetic population assignment

**DOI:** 10.1111/eva.12524

**Published:** 2017-09-14

**Authors:** Emma V. A. Sylvester, Paul Bentzen, Ian R. Bradbury, Marie Clément, Jon Pearce, John Horne, Robert G. Beiko

**Affiliations:** ^1^ Faculty of Computer Science Dalhousie University Halifax NS Canada; ^2^ Marine Gene Probe Laboratory Department of Biology Dalhousie University Halifax NS Canada; ^3^ Department of Fisheries and Oceans St. John's NL Canada; ^4^ Centre for Fisheries Ecosystems Research, Fisheries and Marine Institute Memorial University of Newfoundland St. John's NL Canada; ^5^ Labrador Institute Memorial University of Newfoundland Happy Valley‐Goose Bay NL Canada; ^6^ Northern SE Regional Aquaculture Association Hidden Falls Hatchery Sitka AK USA

**Keywords:** conservation genetics, fisheries management, individual assignment, random forest, SNP selection

## Abstract

Genetic population assignment used to inform wildlife management and conservation efforts requires panels of highly informative genetic markers and sensitive assignment tests. We explored the utility of machine‐learning algorithms (random forest, regularized random forest and guided regularized random forest) compared with *F*_ST_ ranking for selection of single nucleotide polymorphisms (SNP) for fine‐scale population assignment. We applied these methods to an unpublished SNP data set for Atlantic salmon (*Salmo salar*) and a published SNP data set for Alaskan Chinook salmon (*Oncorhynchus tshawytscha*). In each species, we identified the minimum panel size required to obtain a self‐assignment accuracy of at least 90% using each method to create panels of 50–700 markers Panels of SNPs identified using random forest‐based methods performed up to 7.8 and 11.2 percentage points better than *F*_ST_‐selected panels of similar size for the Atlantic salmon and Chinook salmon data, respectively. Self‐assignment accuracy ≥90% was obtained with panels of 670 and 384 SNPs for each data set, respectively, a level of accuracy never reached for these species using *F*_ST_‐selected panels. Our results demonstrate a role for machine‐learning approaches in marker selection across large genomic data sets to improve assignment for management and conservation of exploited populations.

## 1 INTRODUCTION

Genetic assignment of individuals to their source populations is useful for uncovering the spatial distribution of populations and migration patterns (e.g., André et al., 2016) relevant to wildlife management and conservation (Manel, Gaggiotti, & Waples, 2005). For exploited species, assignment tests may be used to monitor population‐specific exploitation, ensuring the maintenance of genetic diversity and improving management practices through the identification of over‐exploited stocks. Assignment tests have been assessed and implemented in commercial fishery species such as herring, *Clupea harengus* L., (Bekkevold et al., 2015), Atlantic cod, *Gadus morhua* L., (André et al., 2016), Chinook salmon, *Oncorhynchus tshawytscha*, (Larson et al., 2014a; Smith, Templin, Seeb, & Seeb, 2005; Templin, Seeb, Jasper, Barclay, & Seeb, 2011) and Atlantic salmon, *Salmo salar* (Bradbury, Hamilton, Rafferty, et al., 2015; Karlsson, Moen, Lien, Glover, & Hindar, 2011). These studies rely on genetic differences among populations to assign individuals to their source populations across large spatial scales (e.g., Bekkevold et al., 2015). Resolution of spatially distinct biological units across fine spatial scales can be difficult as weak genetic divergence may limit the accuracy of assignment tests (Larson et al., 2014a). Developing methods to detect this divergence and improve assignment accuracy may benefit management practices across both large and small geographic scales.

Rapid advances in sequencing and genotyping technologies have enabled the development of large panels of spatially informative single nucleotide polymorphisms (SNPs) from genomewide scans. Markers selected particularly for maximum self‐assignment accuracy are likely to be useful for assignment across both broadscale and small‐scale studies (Larson et al., 2014a); however, the trade‐off between panel size and self‐assignment accuracy often results in panels that, at an adequate performance threshold, are too large to be of practical value for fisheries applications, due to the costs of analysis. Currently, the most widely used methods for SNP selection in ecological research rely on measures of population differentiation (see Helyar et al., 2011; Rosenberg, 2005 for review). Most commonly, SNPs are ranked by fixation index, *F*_ST_ (André et al., 2016; Karlsson et al., 2011; Larson, Seeb, Pascal, Templin, & Seeb, 2014; Larson et al., 2014a; Lemay & Russello, 2015). As a measure of differentiation of populations, *F*_ST_ for SNP selection can be calculated at each locus between subpopulations (pairwise *F*_ST_) or for a metapopulation relative to the overall population (global *F*_ST_; Foll & Gaggiotti, 2006). Although widely used, it is difficult to gauge the applicability of *F*_ST_‐based methods across different study systems because published studies are often biased towards research demonstrating successful self‐assignment. As *F*_ST_‐based methods only consider loci through a single, univariate rank for importance (Brieuc, Ono, Drinan, & Naish, 2015), the overall performance of the selected panel may be limited.

As an alternative, iterative algorithms implemented in the software BELS (Bromaghin, 2008) and genetic algorithms (Topchy, Jain, & Punch, 2004) have been proposed for informative SNP selection (Rosenberg, 2005). Although potentially an improvement for assignment‐focused marker selection, both methods are computationally intensive and BELS lacks consideration of various possible subsets of SNPs (Helyar et al., 2011). In contrast to simple ranking, random forest (RF) is a machine‐learning approach that considers a subset of features or predictive variables (e.g., SNPs) at each node to grow a series of decision trees (Breiman, 2001). In the classification implementation, an individual is assigned to a class (e.g., population), using a bootstrapped sample of these features or loci. Features can be ranked by importance based on the change in classification error affected by the presence or absence of a feature in a subset. The RF algorithm also considers loci in various combinations of subsets, improving the power of the algorithm to rank these features or loci for importance. The increasing popularity of RF in biological research has provided ample evidence to indicate its potential for successful use in population genetics. The regression implementation has been used to select SNPs to predict phenotypes (Brieuc et al., 2015; Bureau et al., 2005; Pavey et al., 2015) and to identify environmental parameters that may have an influence on population structure in landscape genetics (Zhan, 2016). RF classification has been applied as a method of feature selection to predict microbial community structure using phylogenetic and functional trait data (Ning & Beiko, 2015) and to select genes for functionality using microarray data (André et al., 2016; Deng & Runger, 2013; Díaz‐Uriarte & De Andres, 2006; Kursa, 2014); however, to our knowledge it has yet to be applied to SNP selection for population assignment.

Atlantic and Chinook salmon are species that exemplify opportunities, challenges and applications associated with selecting panels of genetic markers for efficient self‐assignment to source populations. Both species are widely distributed, extensively exploited, and of particular conservation concern in parts of their ranges (Bradbury, Hamilton, Dempson, et al., 2015; Bradbury et al., 2016; COSEWIC, 2011; Larson, Seeb, et al., 2014). Both species display natal philopatric behaviour with low rates of straying (Hendry, Castric, Kinnison, & Quinn, 2004; Neville, Isaak, Dunham, Thurow, & Rieman, 2006) and exhibit hierarchical population structure (Bourret, Dionne, Kent, Lien, & Bernatchez, 2013; Templin et al., 2011), making these species ideal candidates for testing assignment efficiency. Despite their philopatric behaviour, fine‐scale assignment of Atlantic and Chinook salmon can be difficult, necessitating novel approaches to detect subtle genetic differences across subpopulations (Greig, Jacobson, & Banks, 2003). Here, we investigate self‐assignment accuracy at fine geographic scales using data obtained from two sources. For Atlantic salmon, we use unpublished data for juveniles sampled from rivers running into Lake Melville, a 3,069 km^2^ marine embayment in Labrador, Canada. Within Lake Melville, food, social and ceremonial (FSC) fishery practices are conducted by Innu First Nations, Inuit (Nunasiavut) and Metis (NunatuKavut) groups and constitute important traditional and recreational harvests (ICES, 2013). An average of 34 tonnes, or approximately 13,200 salmon, are harvested from within and nearby Lake Melville each year (Bradbury, Hamilton, Rafferty, et al., 2015), necessitating a better understanding of stock assessment for management of these populations. For Chinook salmon, we use a published data set (Larson et al., 2014a) with a larger sample size to assess the potential for wider applicability of RF feature (SNP) selection.

Herein, we identify and evaluate various sizes of SNP panels using global *F*_ST_ and three variations of RF: standard, regularized random forest (RRF) and guided regularized random forest (GRRF) (Deng & Runger, 2013). We aim to identify one or more methods for selection of an optimal panel, while comparing the trade‐off between panel size and self‐assignment accuracy across methods and identifying the minimum panel size required to achieve a minimum overall self‐assignment accuracy of 90%. We provide evidence of successful implementation of machine‐learning approaches on a metapopulation scale for site‐by‐site (river) classification to establish a relevant, nonredundant, maximally reduced panel of genetic markers. By testing these novel approaches, we explore methods for capitalizing on large genomic data sets for genetic population assignment, with potential for application across a range of systems.

## 2 MATERIALS AND METHODS

### 2.1 Sampling and genotyping

**Figure 1:**
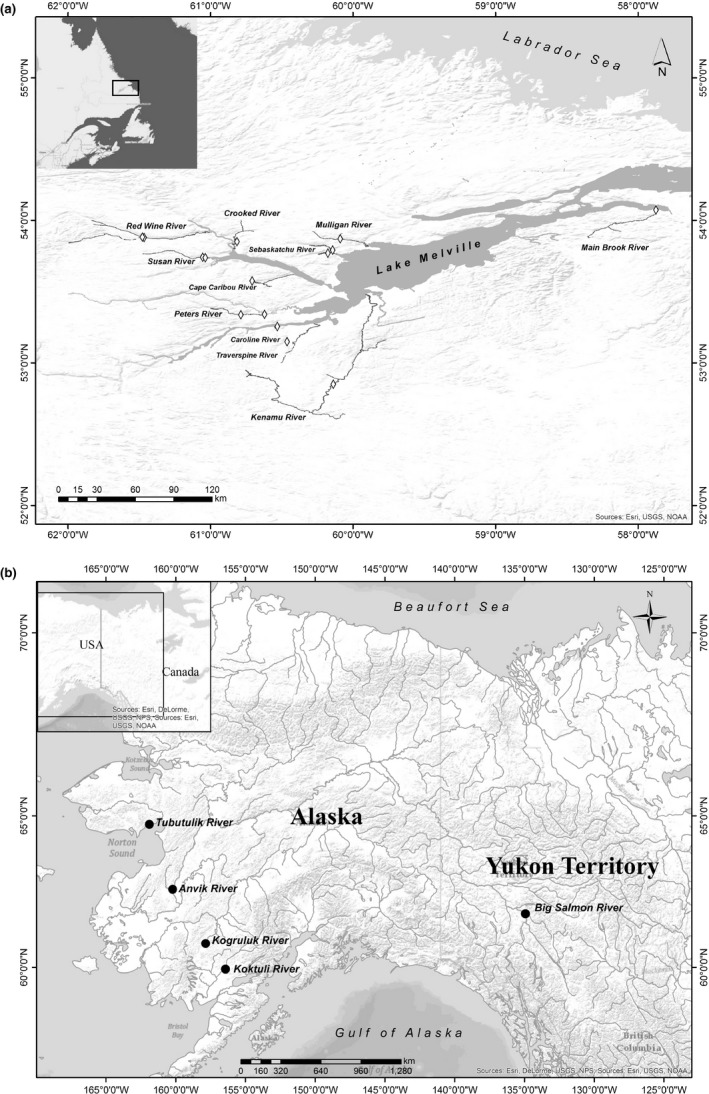
Sampling locations of (a) Atlantic salmon (*Salmo salar)* from Lake Melville, Labrador, Canada and (b) Chinook salmon (*Oncorhynchus tshawytscha*) from western Alaska and the Yukon River. See Table 1 for site coordinates, site ID and sample size for Atlantic salmon sampling. Coordinates for Chinook salmon sampling sites were obtained from Larson et al. (2014a). Maps were created using ArcGIS (ESRI, 2011)

A total of 231 juvenile (parr) Atlantic salmon were sampled from 11 rivers (one to two sites per river) within Lake Melville, Labrador (Table 1, Figure 1), in 2013 and 2014 by electrofishing and angling. Heart samples were collected and placed in 95% ethanol. DNA was isolated using the DNeasy Blood and Tissue kit or DNeasy 96 Blood and Tissue kit (Qiagen, Toronto, ON, Canada) following the manufacturer's protocol, including the optional RNase A treatment. DNA samples were quantified using the Qubit dsDNA HS Assay Kit (Life Technologies, Burlington, ON, Canada) with assays read on a Qubit v2.0 (Life Technologies) or using the Quant‐iT PicoGreen dsDNA Assay Kit (Life Technologies) with assays read on a FLUOStar OPTIMA fluorescence plate reader (BMG Labtech, Ortenberg, Germany). The DNA quality for all samples was verified by agarose gel electrophoresis of 100 ng of extracted DNA, visualized using SYBR Safe (Life Technologies), and documented using a Gel Logic 200 (Kodak, Rochester, NY, USA). Individuals were genotyped using a 220K target, bi‐allelic SNP Affymetrix Axiom array developed by the Centre for Integrative Genetics (CiGene, Ås, Norway). These SNPs were a subset of those in the 930K XHD *Ssa*l array (dbSNP accession numbers ss1867919552–ss1868858426).

**Table 1:** Site locations and sample size for all study collections of juvenile salmon, sampled in 2013 and 2014

River name	Sample size	Site ID	Latitude (N)	Longitude (W)
Cape Caribou River	21	CB	53°32′48,8″	60°36′27,0″
Caroline Brook	20	CL	53°15,232′	60°31,899′
Peters River	21	PR1	53°20′10,4″	60°47′15,3″
		PR2	53°20,345′	60°37,293′
Red Wine River	22	RW1	53°52,764′	61°27,976′
		RW2	53°52,928′	61°28,730′
Susan River	22	SR1	53°44,365′	61°3,275′
		SR2	53°44,184′	61°02,216′
Crooked River	21	CR	53°50,991′	60°48,863′
Kenamu River	22	KE	52°50,952′	60°08,279′
Main Brook River	21	MB	54°04,355′	57°52,374′
Mulligan River	17	MU	53°52,138′	60°05,392′
Sebaskachu River	22	SK1	53°47,397′	60°08,523′
		SK2	53°46,10′	60°10,575′
Traverspine River	22	TR	53°08,853′	60°27,769′

Ten fish were genotyped twice to assess genotyping error rate. Loci with inconsistent calls among replicates were removed from the data set. Loci were then filtered in PLINK v. 1.07 (Purcell et al., 2007) for global minor allele frequency (MAF) below 5%. One locus was also removed for having more than 5% missing data across all sites. Pairwise population *F*_ST_ (Weir & Cockerham, 1984) was calculated using Arlequin v 3.5.2.2 (Excoffier, Laval, & Schneider, 2005). Additional missing genotype data, consisting of 0.08% of the data, were imputed using the function rfImpute in the RandomForest package, using 5,000 trees with all other parameters set to default.

We further reduced our panel for downstream feature selection by removing redundant SNPs and SNPs in linkage disequilibrium using the genepop_toploci function in the R package Genepopedit (Stanley, Jeffery, Wringe, DiBacco, & Bradbury, 2016) at an *R* ^2^ threshold of 0.2 and a minimum global *F*_ST_ of 0.05. Although this is a highly stringent approach, reductions in the data set are helpful both to reduce computational load and to increase consistency of markers across subsets (and therefore confidence in the importance of selected SNPs). As evidence suggests that under linkage disequilibrium, RF performance may be reduced, redundancy in the data set should be considered prior to or during the feature selection process (Meng, Yu, Cupples, Farrer, & Lunetta, 2009; Toloşi & Lengauer, 2011).

Chinook salmon data contained 10,944 SNPs identified through *Sbf1* restriction‐site‐association DNA (RAD) sequencing for 265 adult individuals from five locations: four populations in coastal western Alaska and one in Yukon River (Figure 1b). SNPs were removed from an original pool of 42,351 putative loci, if genotyped in <80% of individuals, and were reduced to one SNP per RAD tag (Larson et al., 2014a). Further, SNPs were filtered for linkage disequilibrium, evidence of paralogous sequences, deviation from Hardy–Weinberg equilibrium and MAFs of <0.05 (Larson et al., 2014a). Data were imputed and filtered for *F*_ST_ and redundancy as described above.

### 2.2 Marker selection

Ideally, assignment analysis with loci selected for population assignment would implement a training/holdout approach, such that the individuals used for marker selection would be different from those used for assignment analysis (Anderson, 2010). Although upward grading bias (over‐estimations of assignment accuracy) is effectively diminished by this approach, a completely independent training and holdout set is often unfeasible due to limitations in sample size. To overcome this, Anderson (2010) proposes a leave‐one‐out strategy where a subset of individuals (training set) are used for locus selection, and all individuals are used to establish a baseline for assignment. However, self‐assignment accuracy is calculated based solely upon the assignment of the individuals in the holdout set. As such, all loci were selected using a subset of individuals. For both data sets, one‐third of the individuals from each site (approximately 7 for Atlantic data and 19 for Chinook data) were randomly selected for all methods of locus selection.

### 2.3 RF‐based SNP selection

#### 2.3.1 Background

For RF classification, measures of importance of each feature can be calculated based on the reduction in accuracy of the model when the feature in question (i.e., SNP) is not included in a subset of features within a tree (Breiman, 2001). Decision trees based on subsets lacking highly informative features will have a higher error or reduced classification accuracy to a known class (i.e., river) when an important feature is removed, compared to an irrelevant marker, the removal of which will result in no reduction in model accuracy. This difference in model accuracy, averaged across decision trees with and without the locus in question is termed the mean decrease in accuracy (MDA). We used this measurement to rank loci based on importance in assignment (classification). Features, or SNPs, with a relatively high MDA will be deemed highly important for accurate classification. As the actual MDA value indicates relative importance in the per cent decrease in accuracy to the model, a strict cut‐off threshold will vary for each data set, depending on how well the population can be inferred by a SNP.

Regularized random forest and GRRF are variations on the RF algorithm designed to address issues with RF, and to optimize features for selection (Deng & Runger, 2013). RRF uses a customizable parameter, the penalty coefficient (λ), which penalizes features at a node when making a classification decision. To be selected for importance and included in the selected panel, a feature must be more informative than the other features in the subset considered at a node as well as those already selected for importance, despite this penalty. As such, RRF is a more stringent application of RF and influences the selected feature set (panel) size. A larger λ (approaching 1) leads to a smaller penalty, resulting in a larger selected panel. Using the minimum regularization (λ = 1) a feature must still be more informative than the already selected features to be included in the subset. Although this additional component to the RF algorithm provides a more stringent approach, the efficacy of RF and RRF may be limited by the number of nodes within the forest that consider a feature for importance to the model. That is, as a locus may not be present in many nodes, it may not be considered for importance often enough to truly inform the selection process, a problem referred to as node sparsity (Deng & Runger, 2013).

Guided regularized random forest addresses node sparsity using an input of importance measures (from a previous RF run, for instance) to weigh each feature. This customizes the algorithm such that the penalty coefficient applied to features of presumably greater importance is less than that applied to features of less importance. GRRF uses an alternative parameter, gamma (γ), to control the weight of the importance score applied to each feature. A larger value of γ (approaching 1) leads to a smaller overall λ and will therefore result in a smaller feature set.

#### 2.3.2 Algorithm application

Data were formatted using a custom R script such that individuals at a given locus were assigned 0, 0.5 or 1, for an individual that is homozygous for the minor allele, heterozygous or homozygous for the major allele, respectively. We ran RF using the R package randomForest (Liaw & Wiener, 2002) on our filtered data sets. To determine our appropriate *ntree* parameter (number of trees), we ran RF using 125, 250, 500, 1,000, 2,000, 4,000 and 8,000 trees, 10 times each. As out‐of‐bag error stabilized at approximately 2,000 trees for both Atlantic and Chinook data, we accepted this as suitable for our analysis (Fig. S2) (Boulesteix, Janitza, Kruppa, & König, 2012). The *m*_try_ parameter (the number of features considered at a node) was tested at default (the square root of the number of features), half default and twice default, as suggested by Liaw and Wiener (2002). Error was lowest at twice default for both Atlantic and Chinook data and was therefore used as such for our analyses. We used a minimum node size (minimum size of terminal nodes or leaves) of five, allowing larger trees to be grown (see randomForest R documentation), with all other parameters set to default (Liaw & Wiener, 2002).

For feature selection, we used five runs of RF, resulting in five separate lists of SNPs ranked by MDA. Panels of various sizes were created by identifying SNPs present in all five lists at 10 ranking levels. These levels were selected to create panels of 40–700 SNPs, after ensuring that each list contained only features with a positive MDA. For example, SNPs consistently ranked within the top 800 loci in all five lists were aggregated to form a consensus panel of 67 SNPs (Table 2).

**Table 2:** Properties of panels selected for assignment analysis by SNP selection method (*F*_ST_ rank, random forest (RF), regularized random forest (RRF) and guided regularized random forest (GRRF) (See Section “2.2”). As RF rank was selected to create panels of target size, panel size column indicates “(Rank) panel size” for RF‐selected panels. See Fig. S3 for intersections of SNPs across methods

Method	Parameter for selection	Parameter value	Panel size Atlantic Salmon	Panel size Chinook Salmon
*F*_ST_	Top ranked	–	60	47
		–	85	65
		–	104	88
		–	130	112
		–	184	134
		–	266	182
		–	344	240
		–	508	384
		–	519	454
		–	670	509
RF	Within (×) rank across all 5 runs	–	(800) 66	(400) 41
		–	(825) 90	(600) 74
		–	(850) 110	(700) 91
		–	(875) 135	(850) 125
		–	(900) 157	(950) 167
		–	(950) 201	(1,000) 216
		–	(1,050) 298	(1,100) 277
		–	(1,200) 435	(1,250) 341
		–	(1,400) 605	(1,400) 437
		–	(1,500) 697	(1,500) 519
RRF	Penalty coefficient (λ)	0.75	51	47
		0.8	83	71
		0.825	114	94
		0.85	140	110
		0.875	180	150
		0.9	275	191
		0.925	336	260
		0.95	515	364
		0.975	604	470
		0.99	710	528
GRRF	Weight of penalty (γ)	0.25	60	47
		0.2	85	65
		0.175	104	88
		0.15	130	112
		0.125	184	134
		0.1	266	182
		0.075	344	240
		0.05	508	384
		0.025	519	454
		0.01	670	509

Regularized random forests and GRRFs were run using the R package RRF (Deng & Runger, 2013). Both methods were run using the same parameters as those used for RF (described above). We tested 10 parameter values for the penalty coefficient (λ) running RRF and 10 parameter values for gamma (γ) when running GRRF (Table 2). Parameters were selected to encompass a range of regularization penalties and to ensure a diversity of panel sizes for individual assignment. A vector of importance measures (MDA scores) determined by a single RF run for feature (SNP) rank was applied for feature weight in GRRF, as described above.

### 2.4 *F*_ST_‐based SNP selection

We tested *F*_ST_ as a method of SNP selection using panels of loci ranked by global *F*_ST_ calculated using the R package Genepopedit (Stanley et al., 2016). To assess the assignment power of various panel sizes of SNPs ranked by *F*_ST_, we created panels of size equal to those established using GRRF for cross‐method comparison (Table 2). To visualize the overlap of SNPs selected across all methods, Venn diagrams were created for the largest panels across all SNP selection methods using Venny 2.1 (Oliveros, 2007–2015).

### 2.5 Individual assignment

The R package Assigner (Gosselin, Benestan, & Bernatchez, 2015) was used to implement “gsi_sim” (Anderson, Waples, & Kalinowski, 2008), to conduct assignment analysis. Assigner is a package developed to run filtering procedures and conduct assignment and mixture analysis with NGS data. By limiting the training set used for marker selection to a subset of individuals as described above, and implementing a LOO cross‐validation method, gsi_sim controls for high grading bias within power analysis without reducing the sample size of the data set. Gsi_sim creates simulations of individual genotypes through bootstrap sampling and assigns these individuals to a population based on the true baseline calculated across all individuals. This is particularly useful for studies with relatively low sample sizes and for fine‐scale studies, where genetic differences in populations are expected to be small. Whitelists, or lists of loci to be considered for assignment, were created from each SNP selection method using custom R scripts for input into Assigner. Although all individuals were used to create the baseline for gsi_sim, only the assignment of the holdout individuals was used to assess self‐assignment accuracy.

Significance of SNP selection method was determined by an ANOVA comparing second degree polynomial models with and without accounting for the SNP selection term. We investigated consistent patterns of incorrect assignment across putative populations (rivers) by observing assignment matrix heatmaps of the smallest panels across all SNP selection methods. We also compared pairwise population *F*_ST_ values to discrepancies in pairwise mismatches (the number of individuals incorrectly assigned across paired populations) between *F*_ST_ rank and GRRF selection methods, to further assess the optimal application of each method. That is, for a given pair of putative populations, the proportion of individuals that were incorrectly assigned from one study site to the other when using GRRF for SNP selection was subtracted from the proportion of individuals incorrectly assigned (within that pair of sites) using *F*_ST_ rank. This allowed us to visualize a preferred method for sites at a given pairwise *F*_ST._

## 3 RESULTS

### 3.1 Genotyping and panel characteristics

Of the original 220K SNPs genotyped for Atlantic salmon, 276 were called inconsistently across samples. Overall genotyping accuracy was >99.8%. After removing these loci and filtering for MAF, 93,058 SNPs remained in the Atlantic salmon data set for further selection. Average global, locus‐specific *F*_ST_ (mean: 0.059, range: 0–0.58) and pairwise population *F*_ST_ ranking across the whole panel (Fig. S1, Table S1) indicated relatively low genetic differentiation. After controlling for linkage disequilibrium and covariance in the panel across all chromosomes, and filtering at a global *F*_ST_ of 0.05, 8,434 nonredundant loci remained in the panel, with *F*_ST_ frequency distribution similar to that observed in the unfiltered data set (Fig. S1). For FST‐based pairwise comparisons of populations, see Table S1. The 10,944 SNP panel accessed for this study (Larson et al., 2014b) was reduced to 2,178 SNPs after filtering at a global *F*_ST_ of 0.05 and linkage threshold of 0.2. For pairwise population *F*_ST_, see Larson et al. (2014a). The size of the panel ranged from 51 to 697 SNPs and 41 to 528 SNPs for the Atlantic salmon and Chinook salmon data sets, respectively (Table 2). Although SNPs were most often selected by only a single selection method, some SNPs were identified by more than one method (Fig. S3). A total of 17 and 32 SNPs were selected by all four SNP selection methods for Atlantic and Chinook salmon, respectively. Overlap in SNPs occurred more often with Chinook salmon data, likely a result of the smaller panel size (2,178 SNPs) relative to the 8,434 SNPs in the Atlantic salmon panel.

### 3.2 Panel performance

#### 3.2.1 Atlantic salmon data

**Figure 2:**
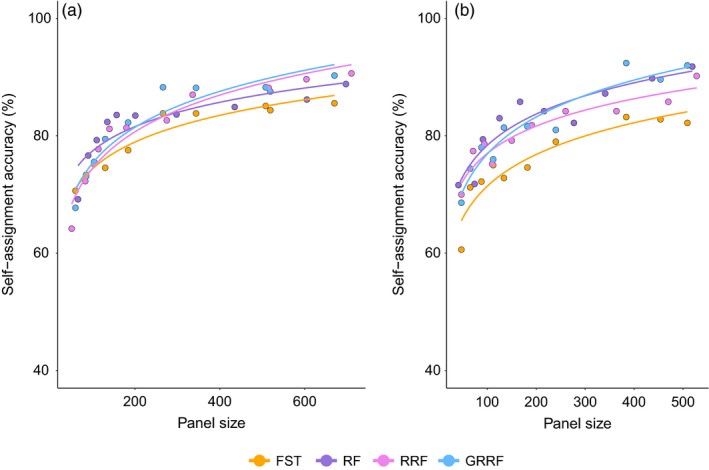
Average, overall self‐assignment accuracy of identified SNP panels (50–700 SNPs) for (a) Atlantic salmon and (b) Chinook salmon (Larson et al., 2014a) calculated across sampling sites. SNP selection method (*F*_ST_ rank, RF, RRF and GRRF) is indicated by colour (see Section “2.2” for more information)

Across panel sizes, we found that panels selected by *F*_ST_ ranking had the lowest self‐assignment accuracy on average (mean = 79.4%, *SE* = 1.8) (Figure 2a). Self‐assignment accuracy for panels selected using RF, RRF and GRRF performed better overall (RF: mean = 81.8%, *SE* = 1.8; RRF: mean = 81.5, *SE* = 2.6; GRRF: mean = 82.1, *SE* = 2.5). An ANOVA comparing the fit of polynomial models with and without considering SNP selection method indicated a marginal significance (*F*_28,37_ = 2.54, *p* < .05). However, this difference varied with panel size. In the smallest panel sizes (50–100 SNPs), *F*_ST_‐ranked panels had better or comparable self‐assignment accuracy with RF‐based panels (Figure 2a). In small‐ to medium‐sized panels (101–200 SNPs), RF‐selected panels performed best (up to 7.8 percentage points for panels of comparable size), while GRRF‐selected panels most often had the highest self‐assignment accuracy in larger panels (>200 SNPs). In all cases, save for the three smallest panel sizes (60, 85 and 104 SNPs), GRRF‐selected panels outperformed *F*_ST_‐selected panels of the same size by a margin of 3.2 to 4.9 percentage points. For smaller panels, RF‐selected panels outperformed *F*_ST_‐selected panels by up to 5%, although the highest accuracy of the smallest panel was 70.64%, observed in the *F*_ST_‐selected panel. A threshold of 90% accuracy overall was achieved only with the largest panels created using GRRF and RRF, which contained 670 and 710 SNPs, respectively.

**Figure 3:**
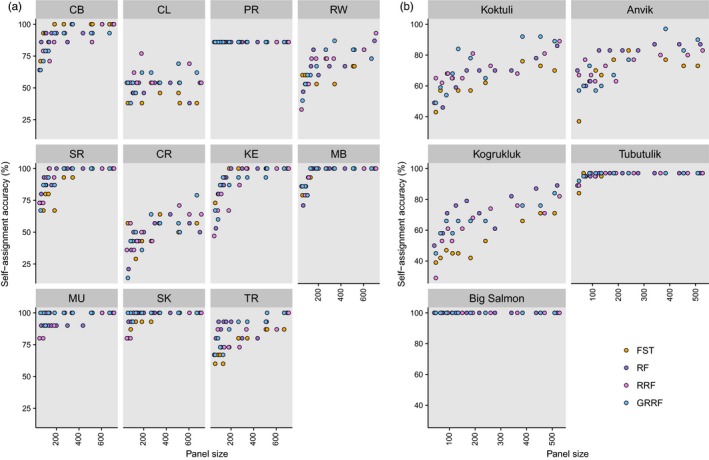
Self‐assignment accuracy of identified SNP panels (50–700 SNPs) across all sampling sites as indicated by site ID (see Table 1) for (a) Atlantic salmon and (b) Chinook salmon (Larson et al., 2014a). SNP selection method (*F*_ST_ rank, RF, RRF and GRRF) is indicated by colour (see Section “2.2” for more information)

We also investigated how self‐assignment varied across sites (Figure 3a). Many sites showed consistently high (above 90%) self‐assignment regardless of SNP selection method, whereas others had a higher frequency of mis‐assignment. In these latter sites (Caroline River and Traverspine River; Red Wine River and Crooked River), the margin in performance between *F*_ST_ and RF‐selected panels widened, in some cases by up to 40 percentage points, as seen in Caroline River (Figure 3a). Some study sites show a higher self‐assignment accuracy with *F*_ST_‐based methods and some with RF‐based methods (Figure 3a). To understand these patterns, we compared pairwise population *F*_ST_ values with the difference in the proportion of mismatches across paired sites between *F*_ST_ and the best performing RF‐based method overall, GRRF (Fig. S4). While we expected that populations with a low pairwise *F*_ST_ value may tend to be more successful with one SNP selection method over another, we did not find consistency across panels. As pairwise *F*_ST_ values increased, these differences shifted towards zero, but at low pairwise *F*_ST_ values, there was no tendency for more mismatches to occur in one method over another (Fig. S4a).

**Figure 4:**
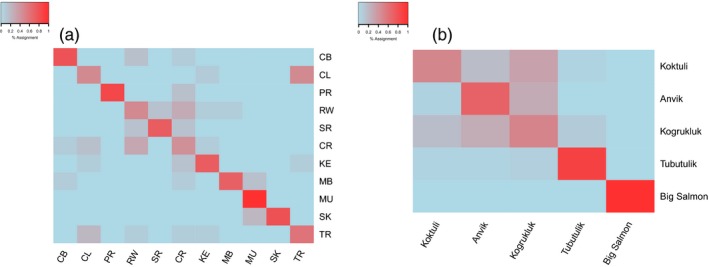
Assignment matrix heatmaps indicating per cent assignment calculated across the best performing panel of the smallest panels (Figure 3). Assignment as determined by (a) *F*_ST_ for Atlantic salmon and (b) RF for Chinook salmon (Larson et al., 2014a). Colour intensity indicates the probability of an individual from a reference population (rows) being assigned to a given population (columns), where red indicates the highest probability and blue the lowest

To identify patterns of mis‐assignment, we created heatmaps demonstrating mis‐assignment from Assigner outputs from the best performing method of the smallest panels (*F*_ST_ and RF for Atlantic and Chinook data, respectively) (Figure 4). We also inspected mis‐assignment across all methods to ensure consistency in observed patterns (Fig. S5). From this, we observed a high rate of mis‐assignment between Red Wine River and Crooked River, and between Caroline River, Traverspine River and, to a lesser degree, Kenamu River. Regardless of the method of SNP selection, we observed that incorrectly assigned individuals from Red Wine River frequently assigned to Crooked River (30.0% of all individuals), and vice versa (35.7% of all individuals). Incorrectly assigned individuals from Caroline River were often assigned to Traverspine River (30.7% of individuals). Although individuals from Traverspine River generally self‐assigned well, incorrectly assigned individuals often assigned to Caroline River (13.3% of all individuals) (Figure 4a). Up to 10% of individuals from Traverspine River and Caroline River incorrectly assigned to Kenamu River, while incorrectly assigned individuals from Kenamu River most often assigned to Traverspine River or Caroline River (up to 13.3%). We also observed consistent self‐assignment of 81% of individuals in Peter's River (Figure 3a). Regardless of panel selection method, the same four individuals mis‐assigned to Crooked River, Red Wine River or Kenamu River (Figure 4, Fig. S5). These consistent patterns in mis‐assignment between geographically proximate sites (Fig. S1a) illustrate the difficulty with population assignment at the finest spatial scales. Although there appears to be some level of genetic divergence between individuals at each of these sites, either computational methods are limited in their ability to detect and fully discern these populations, or they are in fact genetically and behaviourally the same population with higher genetic diversity than nearby populations.

#### 3.2.2 Chinook salmon data

Similar to our findings with the Atlantic salmon data, we found consistently higher self‐assignment accuracy with RF‐based selection methods (RF: mean = 82.7%, *SE* = 2.16; RRF: mean = 80.7%, *SE* = 1.84; GRRF: mean = 81.5%, *SE* = 2.5) compared to *F*_ST_‐selected panels (mean = 75.4%, *SE* = 2.18) (Figure 2b) for the Chinook salmon data set. SNP selection method was found to have a significant effect on the polynomial model (*F*_28,37_ = 4.08, *p* = .001). As observed with the Atlantic salmon data, smaller‐ to medium‐sized panels (up to 200 SNPs) performed best with RF SNP selection (up to 11.2 percentage points for panels of comparable size), while GRRF had the highest self‐assignment accuracy of the larger panels. However, unlike the Atlantic salmon data, *F*_ST_‐selected panels showed reduced self‐assignment accuracy at both small and large panel sizes. GRRF‐selected panels outperformed *F*_ST_‐selected panels of the same size by a margin of 1 to 9.8 percentage points. A 90% self‐assignment accuracy threshold was reached with the largest panels of all RF‐based selection methods, and with a panel of 384 SNPs selected by GRRF at 92.4% overall accuracy.

Self‐assignment accuracy decreased (Figure 3b) and mis‐assignment increased (Figure 4b) among closely associated sites (Anvik River, Koktuli River and Kogrukluk River) with reduced pairwise *F*_ST_ values (Larson et al., 2014a). Larson et al. (2014a) found the lowest genetic divergence between these three rivers, particularly between Kogrukluk River and Koktuli River, as these rivers had the lowest pairwise *F*_ST_ (0.003) and highest occurrence of overlap in a principal component analysis (PCA). Accordingly, we found the highest rate of incorrect assignment occur between these two rivers (Figure 4b). Although *F*_ST_‐selected panels most often had the lowest accuracy, this was not consistent across all sites. As with the Atlantic salmon data, we investigated the relationship between pairwise population *F*_ST_ values and the difference in the number of mismatches occurring between a given pair of populations when using *F*_ST_ values versus the best performing method overall, RF. Although higher pairwise *F*_ST_ is associated with reduced differences between these approaches, there is no indication that outperformance of a particular method is associated with *F*_ST_ (Fig. S4).

## 4 DISCUSSION

Genetic assignment of individuals is becoming central to wildlife management and conservation in many taxa (Reiss, Hoarau, Dickey‐Collas, & Wolff, 2009). Large genomic data sets offer opportunities for increasing assignment power but accurate, practical assignment requires a reduced panel for efficient and cost‐effective analysis (Martinsohn, Ogden, & Consortium, 2009). While a variety of methods currently exist for the identification of targeted panels of markers for population assignment, many are limited by computational load, and naïve algorithms for ranking and selecting SNPs. Machine‐learning algorithms have the potential to address these limitations for wide applicability and success in molecular ecological studies. Here, we demonstrate the use of RF for selecting SNPs for genetic population assignment.

Overall, in both Atlantic salmon and Chinook salmon, we achieved self‐assignment accuracy above 90% for most populations using targeted panels of loci, comparable to or higher than that of broadscale (Bradbury, Hamilton, Dempson, et al., 2015; Bradbury, Hamilton, Rafferty, et al., 2015; Bradbury et al., 2016; Moore et al., 2014; Ozerov et al., 2013) and fine‐scale (Vähä, Erkinaro, Fålkegard, Orell, & Niemelä, 2016) mixed‐stock analyses. Machine‐learning algorithms in contrast to *F*_ST_ rank allow SNPs to be selected based on their relevance directly to the study question, be it correlation with a phenotype (for example, Brieuc et al., 2015) or classification to a reference population. Machine‐learning techniques also consider the importance of loci in combinations with other loci, in contrast to loci selected based solely on individual importance. If combinations of markers perform better than expected given the individual characteristics of each marker, then machine‐learning methods might select relevant markers that would otherwise go undetected. For phenotype–genotype studies, this approach is more likely to consider and identify important loci involved in polygenic traits, which may otherwise be discarded. In a SNP selection study targeting disease indicators (Shah & Kusiak, 2004), a set of 172 SNPs was reduced by 85% with little cost to the performance of the assignment model. It is not surprising then that machine‐learning algorithms may increase the accuracy of population assignment.

### 4.1 Atlantic salmon data

In the Atlantic salmon data set, we observed an improvement of up to 40 percentage points within a given site and up to 7.8 percentage points in overall assignment accuracy, compared to *F*_ST_‐selected panels of similar size. This improvement in self‐assignment accuracy was most apparent in larger panel sizes. In the three smallest panel sizes, *F*_ST_‐selected panels had comparable accuracy to those selected using RF methods. We observed frequent and consistent mis‐assignment in particular sites across SNP selection methods (Figures 3a and 4a). Caroline River and Traverspine River, as well as Red Wine River and Crooked River, showed higher levels of mis‐assignment with each other than most other rivers, although self‐assignment was still higher than would be expected if individuals were randomly assigned to one of these two paired sites (i.e., 50%). This reduction in self‐assignment accuracy likely reflects close genetic relationships or admixing between these neighbouring populations within the same river tributary. Alternatively, this may indicate multiple spawning sites (rivers) for the same population. Pairwise *F*_ST_ values were considerably lower for these pairs of rivers, indicating relatively low genetic divergence (Table S1). We also observed that assignment accuracy within Peter's River rarely deviated from 81%. Across all runs, individuals from Peter's River sampled from the site closest to the river mouth (Figure 1a) were incorrectly assigned to Red Wine River, Crooked River or Susan River. We suspect that there may be genetic structuring occurring within Peter's River or that these individuals are progeny of recent migrants from one or more of these populations. More samples to detect population structure within these rivers may indicate the presence of distinct upstream and downstream populations within Peter's River, or other rivers with natural barriers influencing within‐stream population structure. Although our study revealed clear patterns of mis‐assignment in pairs, it is likely that patterns of incorrect assignment in other natural systems may be more complex (Vähä et al., 2016), particularly when assigning to a greater number of sites (Moore et al., 2014) or if the subpopulations in question are less genetically divergent. For such studies, GRRF or other modified machine‐learning approaches may be well suited to SNP selection for accurate overall assignment accuracy, as shown by the successful application in the present study.

### 4.2 Chinook salmon data

In Chinook salmon, our applications of RF‐based methods to a large (10,944 SNPs), published data set (Larson et al., 2014a), provided further evidence of the usefulness of RF feature selection. RF‐selected panels had consistently higher self‐assignment accuracy compared to those selected by *F*_ST_ ranking. Using a panel of 39 SNPs developed from expressed sequence tags, Larson et al. (2014a) obtained an overall accuracy of 54.4% using a LOO approach, comparable to our smallest *F*_ST_‐ranked panel of 47 SNPs, with an overall accuracy of 60.6% (Figure 2b). However, the smallest RF‐based panels resulted in overall self‐assignment accuracy of 71.6%, 70.0% and 68.6% for RF, RRF and GRRF, respectively (Figure 2b). Self‐assignment accuracy of the largest panel (509 SNPs) using GRRF was comparable to that achieved using all 10,944 SNPs (Larson et al., 2014a) (92.0% and 96.4%, for the 509 SNP panel and 10,944 SNP panel, respectively). Comparable self‐assignment accuracy (above 90%) was reached using a panel of 500 multi‐SNP (haplotype) loci (McKinney, Seeb, & Seeb, 2017) selected based on FST rank with individuals assigned using GSI_sim. In this study, McKinney et al. (2017) combined Koktuli River and Kogrukluk River into a single group for mixture analysis and individual assignment. That we achieved a similar level of self‐assignment accuracy with single‐SNP panels of equal or lesser size without combining sampling locations speaks to the predictive power of RF‐based methods for marker selection. Populations with the lowest self‐assignment accuracy (Anvik River, Kogrukluk River and Koktuli River) (Figure 3b and 4b) were consistent with those found to be the least divergent, with the lowest pairwise FST (0.003–0.006) and high degree of overlap in a PCA analysis (Larson et al., 2014a). While *F*_ST_‐selected panels had the lowest accuracy for Kogrukluk River and Koktuli River, this disparity was reduced in Anvik River.

### 4.3 Overall findings

Random forest methods often outperformed the *F*_ST_‐based method; however, the Atlantic and Chinook salmon data showed discrepancies in the optimal method of SNP selection for each site. By comparing pairwise *F*_ST_ with the difference in the number of mismatches between paired populations when using the best RF‐based method and *F*_ST_ for SNP selection, we hoped to elucidate these findings. However, we did not find strong evidence that either of these methods performs better under certain conditions of population divergence (Fig. S4).

Across all analyses, we often observed fluctuations in self‐assignment accuracy. There are many instances of accuracy decreasing with increasing panel size, even when markers were selected using the same method (Figures 3 and 4). Using a simulated baseline based on a subset of SNPs for individual assignment leaves room for noise and minor fluctuations depending on the SNPs used for assignment. Increasing panel size would not always increase accuracy if less‐informative SNPs are also included in the panel. Although our methods aim to select the most informative SNPs, those selected for classification based on the training set of individuals may not be informative for assignment when applied to the holdout individuals.

Although there was little difference observed between the three RF‐based methods, in both data sets RF‐selected panels had higher assignment accuracy in small‐ to medium‐sized panels, while GRRF often outperformed other SNP selection methods in the largest panels. This reduction in RF accuracy may be due to our applications of the RF approach. As we aggregated SNPs across five lists ranked by MDA, loci common across all lists at a lower rank may not be any more informative than those already included in the smaller panels and will therefore contribute little to assignment accuracy. Conversely, GRRF continues to apply a penalty to SNPs regardless of panel size and thus selects SNPs that continue to contribute to the overall informativeness of the panel. We tested RRF and GRRF in addition to the basic RF approach to address the possible risk of node sparsity and to demonstrate the potential benefits of more stringent approaches. The easy implementation and customizable parameters for panel size selection speak to the usability of these algorithms for subset selection. One additional benefit of GRRF is the customizable weighting of loci. We applied importance scores from a previous RF run to apply a nonuniform weight to the error penalty for each SNP. However, these scores could reflect additional information, such as location within known genes or importance to a phenotypic trait to allow for functional importance of loci to be considered in the SNP selection process. As such, we believe the comparison of all three approaches informs future use across genetic‐based disciplines.

Sampling juveniles at spawning sites of anadromous fish increases the possibility of including siblings within the sample. Although this might inflate our estimates of self‐assignment accuracy for Atlantic salmon, purging the data set of siblings may actually reduce population estimates, depending on the severity of sibling removal (Waples & Anderson, 2017). The ideal threshold to remove individuals can be difficult to determine and varies for different systems and data sets (Waples & Anderson, 2017). Further, this bias would be consistent across SNP selection methods and does not detract from the benefits of machine‐learning methods for SNP selection. The improved self‐assignment accuracy obtained with RF methods for a larger sample of adult Chinook salmon (Larson et al., 2014a) demonstrates a wider range of the applicability of this approach.

We applied RF feature selection to populations under a hierarchical genetic structure. Further tests of these methods may reveal that the applicability of RF is limited to highly structured populations under this type of hierarchical model. However, we demonstrate that within these populations of low differentiation (low pairwise *F*_ST_), there is potential to develop these methods for further research. The resolution achieved using a single, small panel of SNPs for river‐scale assignment offers new opportunities to improve fisheries management techniques. Ozerov et al. (2013) found that to distinguish populations of Atlantic salmon to a comparable (90%) accuracy, different sets of up to 150 SNPs were required to classify mixtures of individuals, depending on the populations in question. Although it is possible that there is some upward grading bias in our study, we applied the combined training‐holdout and LOO method proposed by Anderson (2010) to reduce overestimation of self‐assignment accuracy that might otherwise occur with relatively low sample sizes.

As we investigated overall assignment using a single panel at a time, we cannot be sure how each SNP in the subset distinguishes individuals within a river. The low degree of overlap across RF runs (Table 2, Fig. S3) indicates high variation in the RF ranking process. This is expected due to the randomness associated with considering subsets of features within each tree, but may be indicative of noise that must be filtered by the RF algorithm. Although the proportion of SNPs present in all five runs increases with increasing rank (Table 2), an adapted algorithm to increase consistency may also improve results. Though outside of the scope of the present study, investigating the potential for a deterministic approach could provide insight into the underlying genetic differentiation between certain populations and the process of feature ranking in RF. Our findings support the use of stringent applications of RF for feature selection in a wildlife management context, such that a reduced panel may be established to allow for individual assignment to natal rivers. With this improvement in accuracy, these methods could be used to inform management policies to reduce exploitation of particular subpopulations. This study highlights the need for further investigation of machine‐learning techniques, such as RF, that may be valuable for a range of ecological studies.

## 5 SUMMARY

Large genomic data sets offer new potential for resolving population structure and improving assignment power and accuracy. However, the identification of informative panels of loci from these large data sets remains a challenge. Here, we apply a machine‐learning approach, RF and variations of RF as a useful method of feature selection across large SNP panels. These methods may be used for further application towards selecting relevant panels for monitoring stock and assessing wildlife management strategies.

## DATA ARCHIVING STATEMENT

Data and custom R scripts used for this study are available from the Dryad Digital Repository: https://doi.org/10.5061/dryad.93h33.
