# Supplementary material for: Applications of random forest feature selection for fine‐scale genetic population assignment
Source: Evol Appl. 2017 Sep 14;11(2):153–65. doi: 10.1111/eva.12524 (PMC5775496; doi:10.1111/eva.12524)
Supplement: Supplementary file 1 [file EVA-11-153-s001.pdf]

Supporting Information

Table S1: Pairwise population F_{ST} (bottom diagonal) and p-values (top diagonal) calculated using 1000 interations in Arlequin 3.5.2.2 (Excoffier et al. 2005). P-values of zero indicate values < $e10^{-6}$.

	CB	CL	PR	RW	SR	CR	KE	MB	MU	SK	TR
CB	-	0	0	0	0	0	0	0	0	0	0
CL	0.03435	-	0	0	0	0	0	0	0	0	0.00098
PR	0.13131	0.13626	-	0	0	0	0	0	0	0	0
RW	0.02427	0.03304	0.12299	-	0	0.00488	0	0	0	0	0
SR	0.03733	0.0452	0.13336	0.0257	-	0	0	0	0	0	0
CR	0.01992	0.02736	0.12003	0.00354	0.02315	-	0	0	0	0	0
KE	0.0253	0.02207	0.12588	0.02628	0.0379	0.02347	-	0	0	0	0
MB	0.05302	0.06145	0.15128	0.04663	0.05774	0.04212	0.05829	-	0	0	0
MU	0.06121	0.06828	0.15461	0.05537	0.06514	0.05036	0.06761	0.03697	-	0	0
SK	0.07608	0.08446	0.16597	0.07009	0.07911	0.06477	0.08092	0.05637	0.03127	-	0
TR	0.03682	0.0106	0.13855	0.03651	0.04923	0.03347	0.01772	0.06956	0.07567	0.09025	-

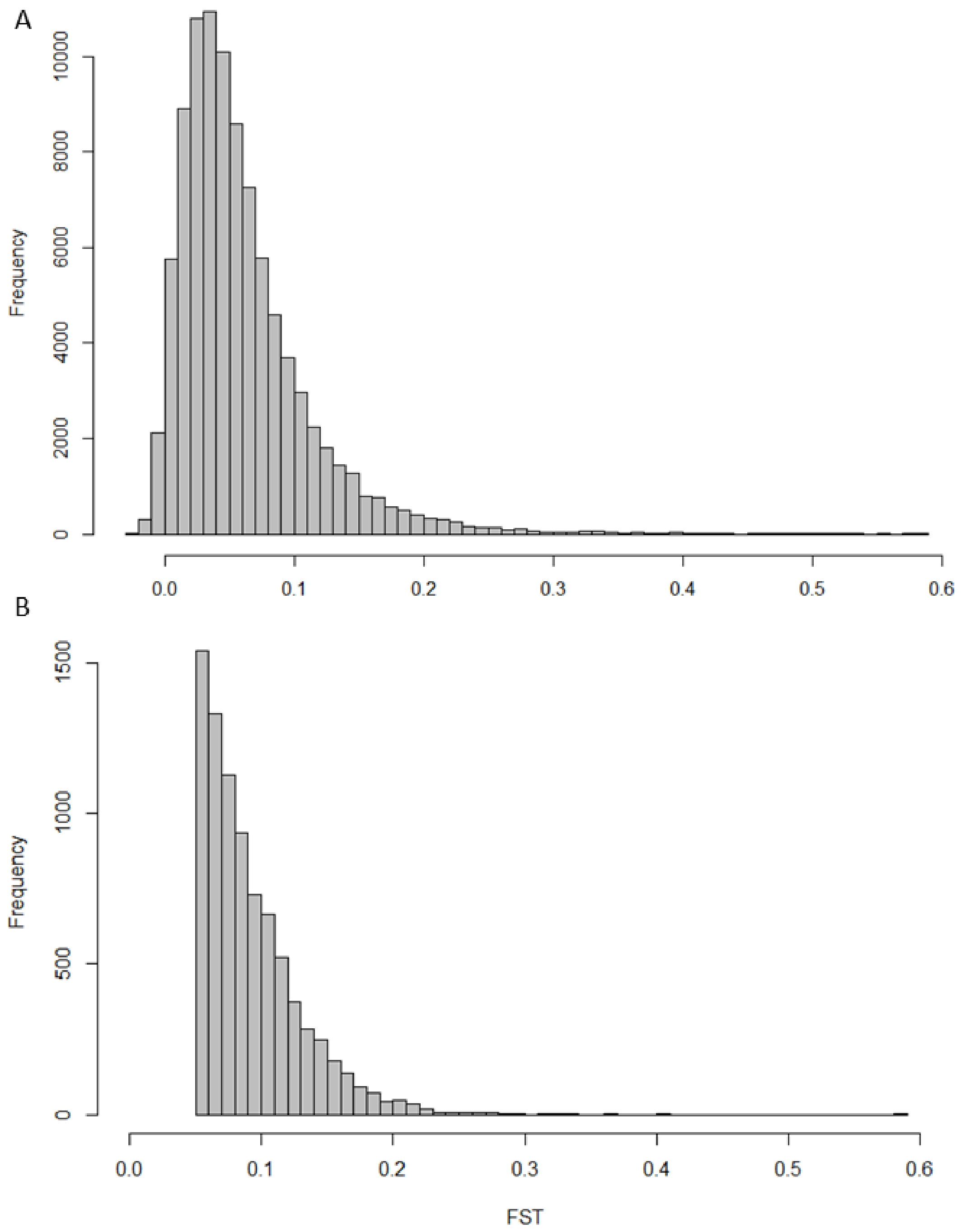

Figure S1. Frequency distribution of global F_{ST} (A) across all loci after initial filtering (93,058 SNPs) and (B) after filtering for redundancy (R^2 linkage threshold (based on correlation between loci) of 0.2) and F_{ST} (threshold of 0.05) in Genepopedit (Stanley et al. 2016).

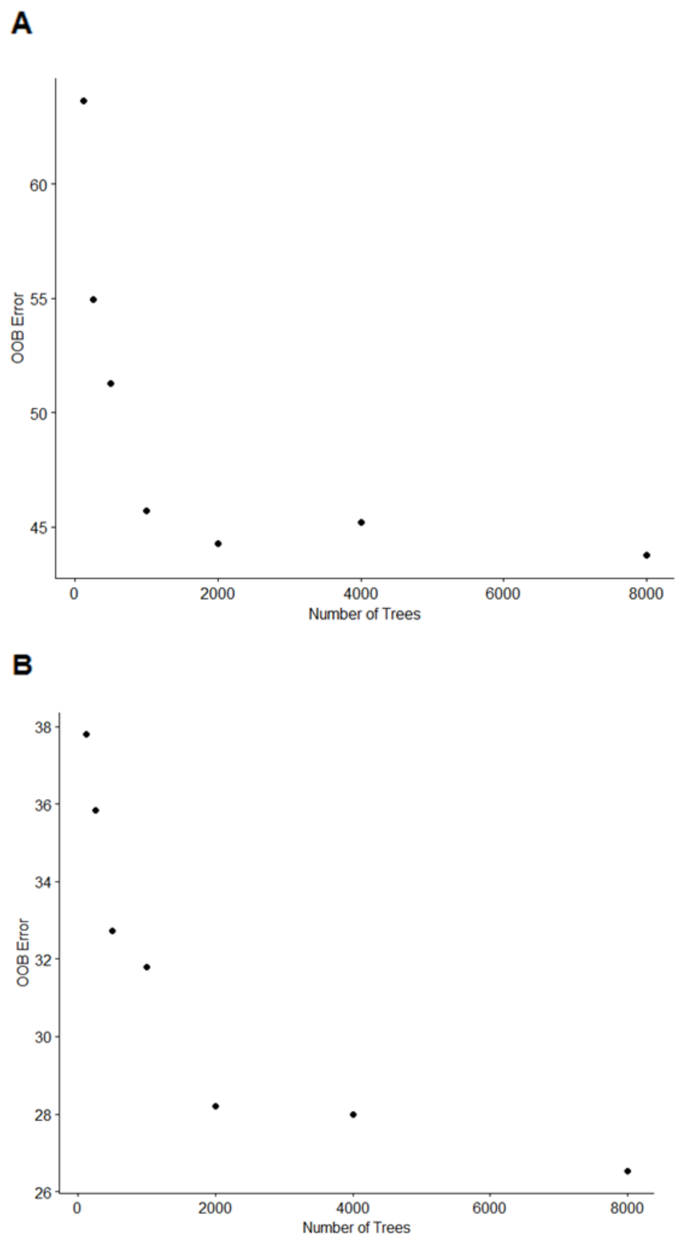

Figure S2. Average out-of-bag (OOB) error calculated using 125, 250, 500, 1000, 2000, 4000 and 8000 trees with 10 runs each to determine appropriate number of trees used for downstream RF analyses for (A) Atlantic salmon data and (B) Chinook salmon data (Larson et al. 2014a).

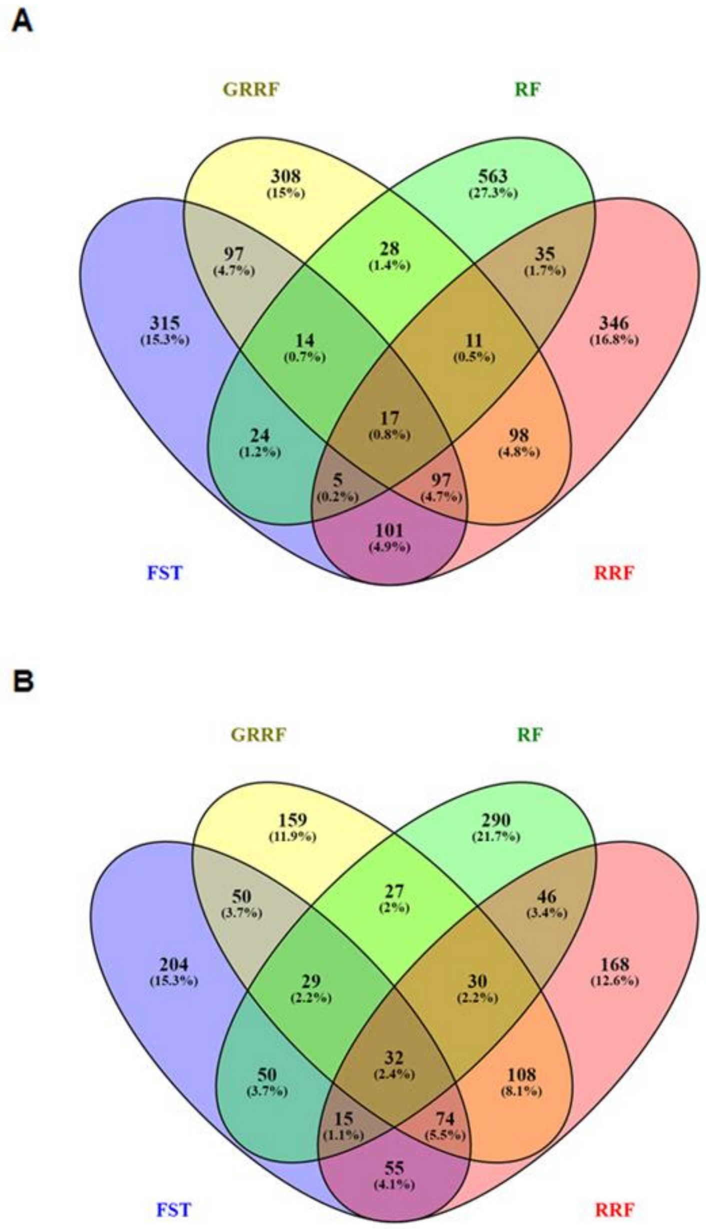

Figure S3. Overlap of SNPs from largest panels created using F_{ST}, Random Forest (RF), Regularized Random Forest (RRF) and Guided Regularized Random Forest (GRRF) for (A) Atlantic salmon data and (B) Chinook salmon data (Larson et al. 2014a). See Table 2 of manuscript for panel information. Diagrams created using Venny 2.1.

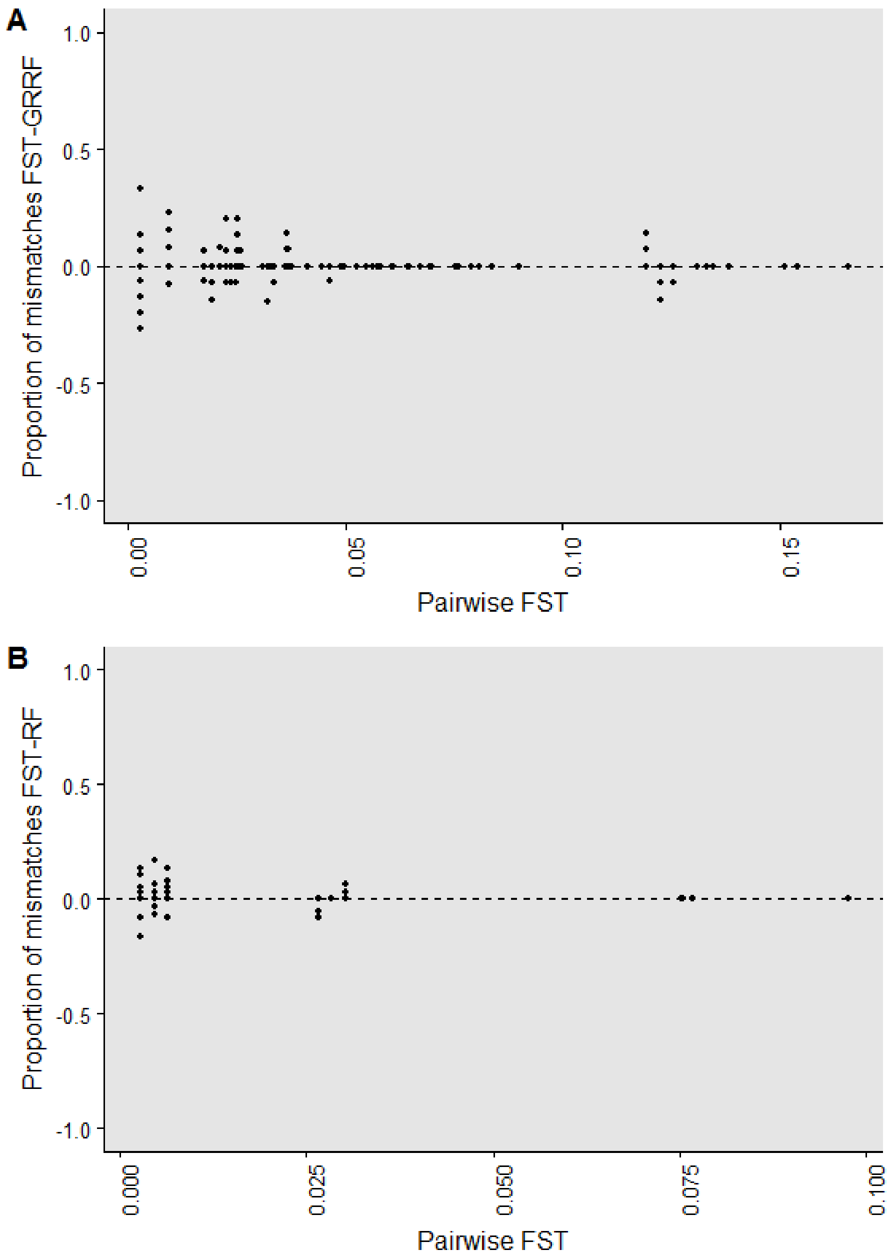

Figure S4. Difference between F_{ST} and best overall RF-based SNP-selection method showing proportion of individuals from one study location incorrectly assigned to an alternative location, sorted by pairwise population F_{ST} for (A) Atlantic salmon, comparing F_{ST} and GRRF and (B) Chinook salmon (Larson et al. 2014a), comparing F_{ST} and RF.

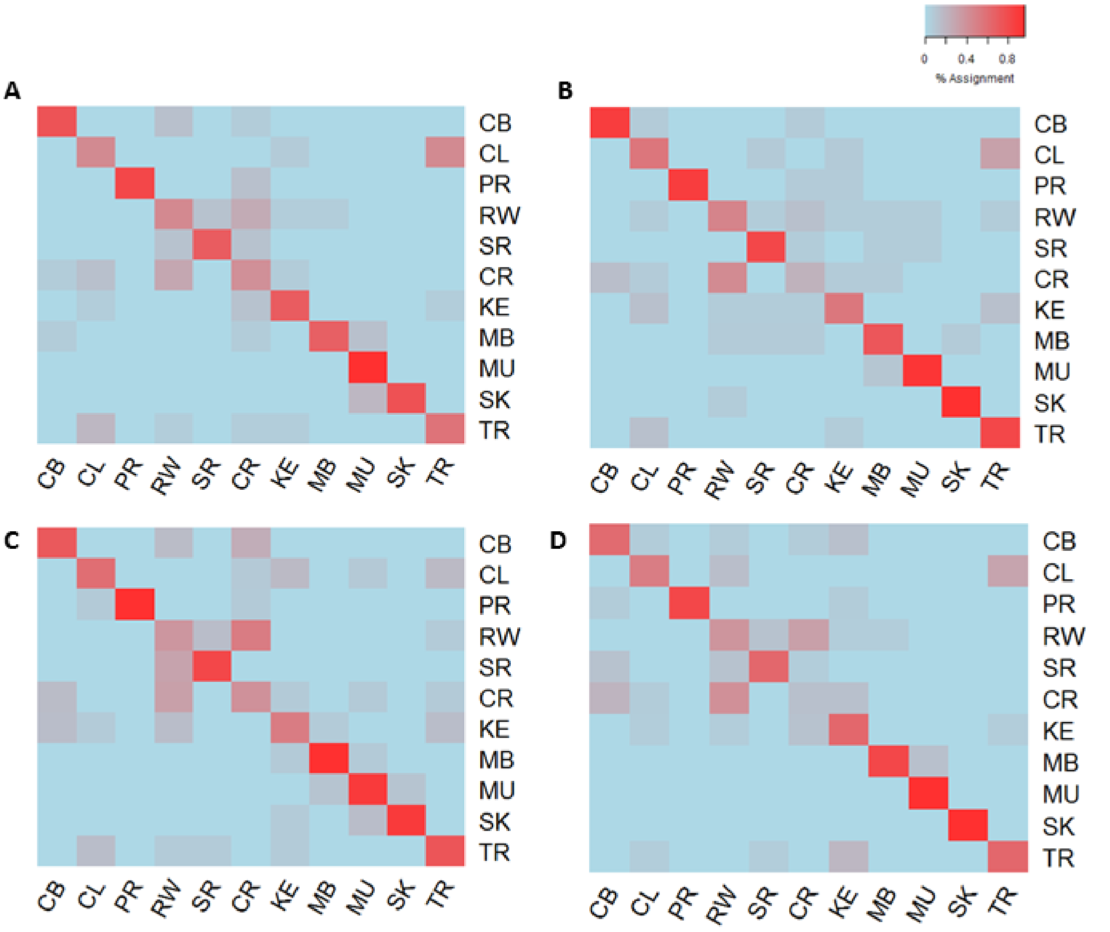

Figure S5. Assignment matrix heat maps indicating average percent assignment of Atlantic salmon data calculated across the smallest panels (51-66 SNPs) established using (A) FST rank, (B) RF, (C) RRF and (D) GRRF. Colour intensity indicates probability of an individual from a reference population (rows) being assigned to a given population (columns).
